# Supplementary material for: Radiationless mechanism of UV deactivation by cuticle phenolics in plants
Source: Nat Commun. 2022 Apr 4;13:1786. doi: 10.1038/s41467-022-29460-9 (PMC8979964; doi:10.1038/s41467-022-29460-9)
Supplement: Supplementary file 1 — Supplementary Information [file 41467_2022_29460_MOESM1_ESM.pdf]

SUPPLEMENTARY INFORMATION

Radiationless mechanism of UV deactivation by cuticle phenolics in plants

Ana González Moreno[1], Abel de Cózar[2,3*], Pilar Prieto[4], Eva Domínguez[5] and Antonio Heredia[1*]

[1]Instituto de Hortofruticultura Subtropical y Mediterránea La Mayora, Universidad de Málaga - Consejo Superior de Investigaciones Científicas Departamento de Biología Molecular y Bioquímica, Universidad de Málaga, E-29071 Málaga, Spain.

[2]Departamento de Química Orgánica I / Kimika Organikoa I Saila, Facultad de Química / Kimika Fakultatea, Universidad del País Vasco / Euskal Herriko Unibertsitatea (UPV/EHU) and Donostia International Physics Center (DIPC), P. K. 1072, 20018 San Sebastián – Donostia, Spain.

[3]Ikerbasque, Basque Foundation for Science, Plaza Euskadi 5, 48009 Bilbao, Spain.

[4]Departamento de Inorgánica, Orgánica y Bioquímica. Facultad de Ciencias y Tecnología Química-IRICA, Universidad de Castilla-La Mancha, 13071 Ciudad Real, Spain.

[5]Instituto de Hortofruticultura Subtropical y Mediterránea La Mayora, Universidad de Málaga - Consejo Superior de Investigaciones Científicas, Departamento de Mejora Genética y Biotecnología, Estación Experimental La Mayora, Algarrobo-Costa, E-29750 Málaga, Spain.

SUPPLEMENTARY INFORMATION CONTENTS:

Supplementary Tables 1-3. Supplementary Figures 1-20.

Supplementary Table 1. Total electronic energies (E, in a.u.),[a,b,c] zero point correction of the energy[d] (ZPCE), thermal correction to Gibbs free energies[d] (TCGFE, in a.u.), and number of imaginary frequencies[e] (NIMAG) of all stationary points discussed in the text for the solvated system (s). Single point energy calculations at CASSCF(12,11)(PCM,methanol)[b] and CASPT2(PCM,methanol)[c] levels (values between brackets and square brackets, respectively) of optimized DFT(PCM,methanol)[a] geometries are also included.

Structure	E	ZPCE	TCGFE	NIMAG(ν)
s-trans-S_0	-573.309676[a]<br>(-570.130972)[b]<br>[-572.393289][c]	0.154841	0.117400	0
s-trans-S_1	-573.174169 [a]<br>(-570.021290)[b]<br>[-572.221907][c]	0.151628	0.113174	0
s-TS-S_0	-573.219446 [a]<br>(-570.028202)[b]<br>[-527.297158][c]	0.151522	0.114539	1 (-1326.952)
s-TS-S_1	-573.171698 [a]<br>(-570.032044)[b]<br>[-572.230115][c]	0.151229	0.115135	1 (-117.2552)
s-cis-S_0	-573.300956 [a]<br>(-570.1195684)[b]<br>[-572.387506][c]	0.155324	0.117220	0
s-cisS_1	-573.209625 [a]<br>(-570.018820)[b]<br>[-572.265923][c]	0.245532	0.208307	0

[a]Computed at wb97XD(PCM, methanol)/6-31+G(d,p) level. [b]Computed at CASSCF(12,11)(PCM,methanol)/6-31+G(d,p)//wb97XD(PCM, methanol)/6-31+G(d,p) level. [c]Computed at CASPT2(PCM,methanol)/cc-pVTZ // wb97XD (PCM, methanol) /6-31+G(d,p) level. [d]Computed at 298.15 K at wb97XD(PCM, methanol)/6-31+G(d,p) level. [e]If NIMAG=1, the imaginary frequency (ν), in parenthesis, is given in cm^{-1}.

Supplementary Table 2. Total electronic energies (E, in a.u.),[a,b,c] zero point correction of the energy[d] (ZPCE), thermal correction to Gibbs free energies[d] (TCGFE, in a.u.), and number of imaginary frequencies[e] (NIMAG) of all stationary points discussed in the text for the solid phase (sp). Single point energy calculations at CASSCF(12,11)[b] and CASPT2[c] levels (values between brackets and square brackets, respectively) of optimized DFT[a] geometries are also included.

Structure	E	ZPCE	TCGFE	NIMAG(ν)
sp-trans-S_0	-573.294364 [a] (-570.128604)[b] [-572.371889][c]	0.155068	0.117249	0
sp-trans-S_1	-573.141973 [a] (-570.007745)[b] [-572.207243][c]	0.151551	0.113651	0
sp-TS-S_0	-573.194777 [a] (-570.026006)[b] [-572.266035][c]	0.152356	0.116325	1 (-706.0593)
sp-TS-S_1	-573.140228 [a] (-570.046224)[b] [-572.192997][c]	0.152028	0.115926	1 (-107.3916)
sp-cis-S_0	-573.288377 [a] (-570.119913)[b] [-572.364922][c]	0.155642	0.118266	0

[a]Computed at wb97XD/6-31+G(d,p) level. [b]Computed at CASSCF(12,11)/6-31+G(d,p)//wb97XD/6-31+G(d,p) level. [c]Computed at CASPT2/cc-pVTZ // wb97XD /6-31+G(d,p) level. [d]Computed at 298.15 K at wb97XD/6-31+G(d,p) level. [e]If NIMAG=1, the imaginary frequency (ν), in parenthesis, is given in cm^{-1}.

Supplementary Table 3. S_0 and S_1 electronic energies (E, in a.u.) of solvated (s) and solid phase (sp) conical intersections (CI) discussed in the text computed at CASSCF(12,11)(PCM,methanol)/6-31+G(d,p), CASSCF(12,11)/6-31+G(d,p) levels.[a] Single point energies computed at CASPT2(PCM,methanol)/cc-pVTZ and CASPT2/ cc-pVTZ levels are also included (values between brackets).[b]

Structure	E S_0	E S_1
s-CI	-570.033779[a] (-572.287933)[b]	-570.031425[a] (-572.266781)[b]
s-CI’	-570.030673[a] (-572.274254)[b]	-570.065870[a] (-572.268613)[b]
sp-CI	-570.038027[a] (-572.253122)[b]	-570.038648[a] (-572.254544)[b]

Results show slightly differences between the CASSCF and CASPT2 energetic surfaces due to consideration of dynamic electron correlation in the solvated scenario. As a result conical intersections optimized at CASSCF(12,11) (i.e. points in the energetic surface were S_0 and S_1 electronic states show similar energy) display higher S_0–S_1 energetic differences when performing CASPT2 single point energy calculations. In order to account for that discrepancy, we have also included s-CI’ in Supplementary Table 3, that corresponds to S_0–S_1 minimum energy paths computed at CASPT2 level. Remarkably, the geometry of that conical intersection in CASPT2 space (s-CI’) is quite similar to s-CI with a dihedral angle of $\theta = 91.0^{o}$ (See Supplementary Figure 7).

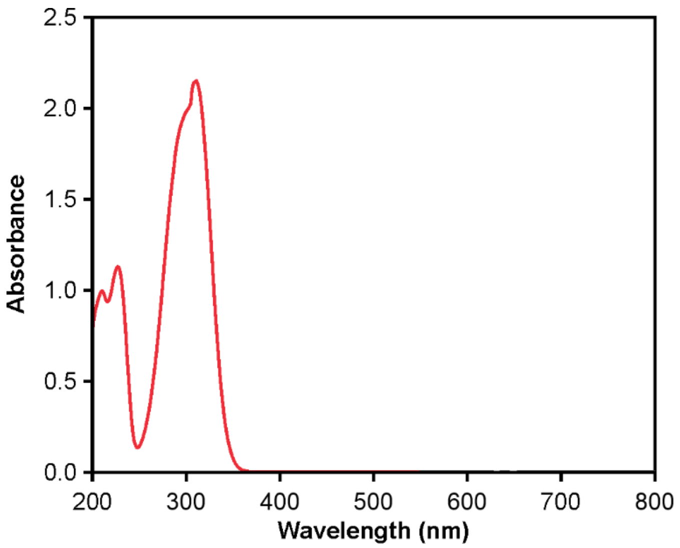

Supplementary Figure 1. Absorbance spectra of *p*-coumaric acid in methanol.

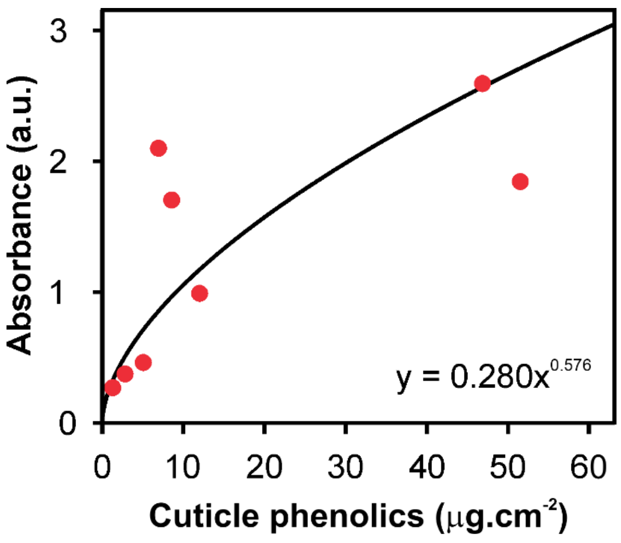

Supplementary Figure 2. Scatter plot of cuticle absorbance in the UV-B range and amount of cuticle phenolics per surface unit. Average cuticle absorbance in the UV-B region is represented. Curvilinear regression fit, solid line, is shown (Determination coefficient R^2 = 0.699, F-test =13.909 and p= 0.01).

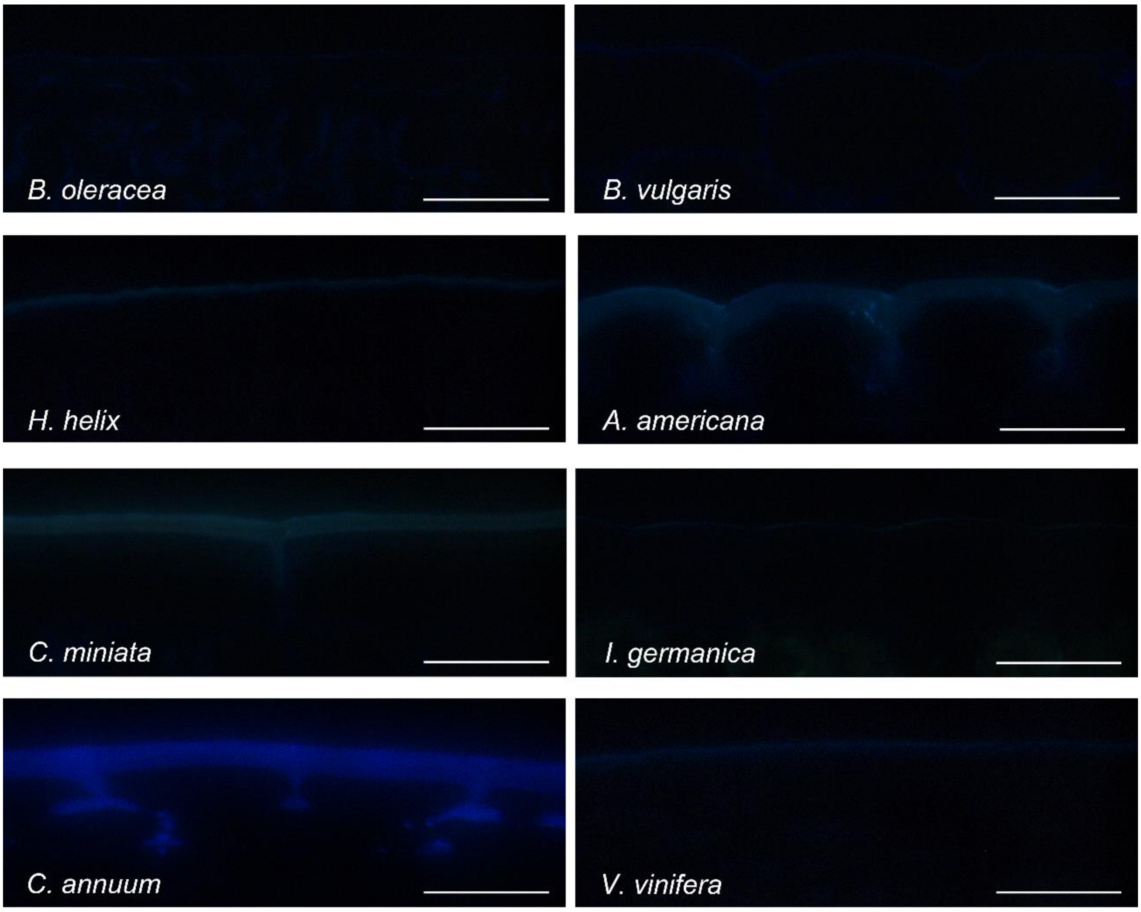

Supplementary Figure 3. Autofluorescence of epidermal cross-sections from various species. Autofluorescence after UV excitation of *Brassica oleracea*, *Beta vulgaris*, *Hedera helix*, *Agave americana*, *Clivia miniata* and *Iris germanica* leaf epidermis. *Capsicum annuum* and *Vitis vinifera* mature fruit epidermis. Scale bar, 50 μm. n=3 biologically independent samples for each species.

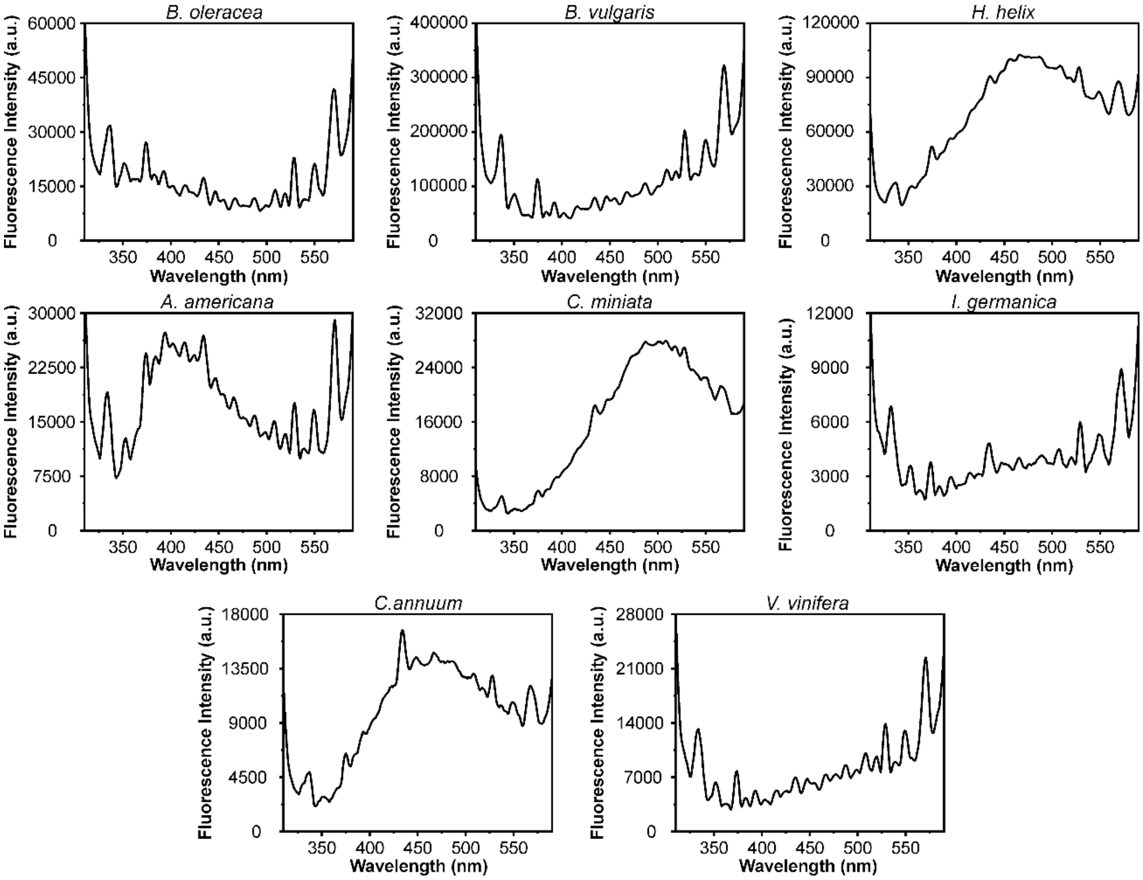

Supplementary Figure 4. Fluorescence emission spectra of isolated cuticles. Autofluorescence after UV excitation at 300 nm of *Brassica oleracea*, *Beta vulgaris*, *Hedera helix*, *Agave americana*, *Clivia miniata* and *Iris germanica* leaf cuticles and *Capsicum annuum* and *Vitis vinifera* mature fruit cuticles.

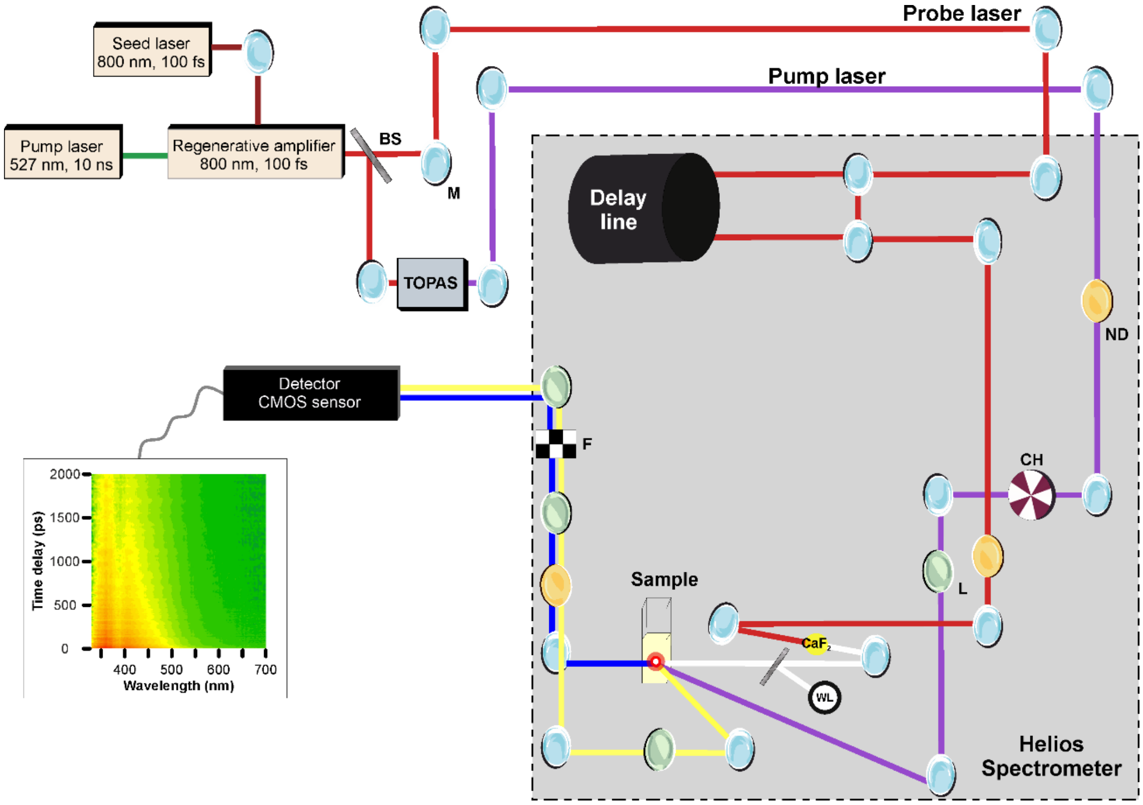

Supplementary Figure 5. Schematic layout of the set up for transient measurements. Coloured lines represent lasers at different wavelengths. **BS:** Beam Splitter; **M:** Mirror; **ND**: Neutral Density filter; **CH:** Chopper; **L:** Lens; **F:** filter; **WL:** parallel detection of generated white light used as control. **CaF_2:** non-lineal mirror that generates white light. Two detection modes are represented, yellow and blue lines. Blue line represents the transmission detection used for liquid samples and yellow beam the reflection mode employed for solid samples. A red amplified laser is split into the two lasers needed for transient experiments, pump (reflected beam) and probe lasers (transmitted beam). Pump laser crosses the TOPAS, where the excitation wavelength is selected. Then, both lasers are conducted to the Helios spectrometer. When the pump reaches the sample, molecules susceptible to be excited at that wavelength experiment an electronic transition. A chopper is employed to alternatively obtain signals of the excited and non-excited sample and therefore, to acquire the difference absorption maps

(transient maps). The probe laser generates white light through a non-linear mirror which allows the visualization of the processes taking place in the excited state within a time range below nanoseconds. The probe is collected by an optical fiber to the detector following transmission or reflection mode depending on the nature of the sample, liquid or solid respectively.

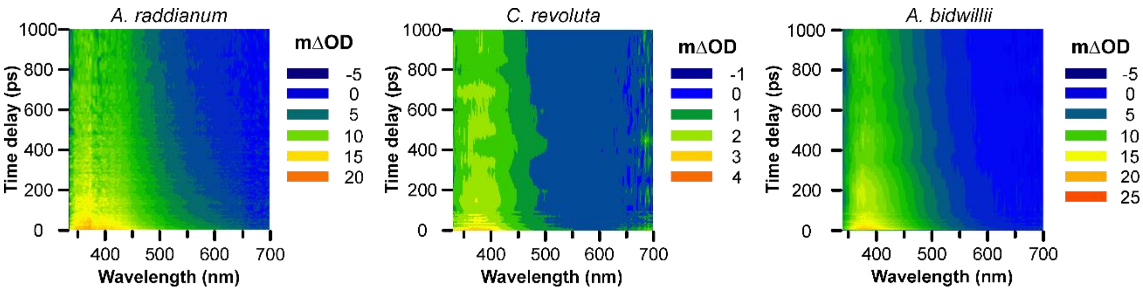

Supplementary Figure 6. Transient absorption spectra (TAS) of different leaf epidermises. Heat maps of *Adiantum raddianum*, *Cycas revoluta* and *Araucaria bidwillii* displaying differences in optical density (mΔOD) with time and wavelength after a pump pulse of 300 nm.

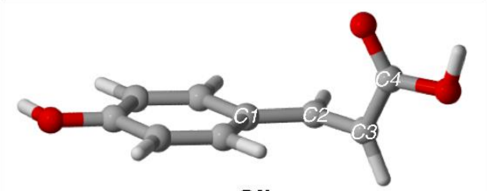

s-CI’

$\theta_{C1\text{-}C2\text{-}C3\text{-}C4}$ = 91.0º

Supplementary Figure 7. Geometry of conical intersection s-CI’ computed at CASPT2(PCM,methanol)/cc-pVTZ level of theory. See comments in Supplementary Table 3.

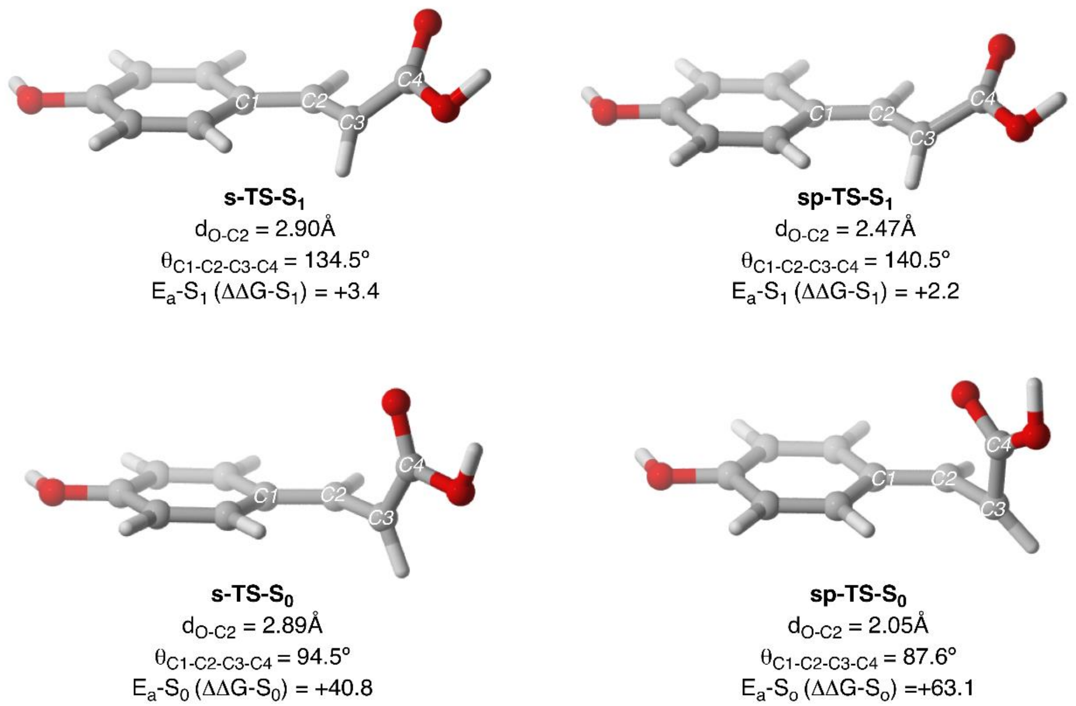

Supplementary Figure 8. Main geometrical features of transition states in the basal state S_0 and in electronic excited state S_1 computed at MP2(PCM, methanol)/6-31+G(d,p)//wb97XD(PCM, methanol)/6-31+G(d,p) or MP2)/6-31+G(d,p)//wb97XD/6-31+G(d,p) level. Activation Gibbs free energies (ΔΔG) are in kcal mol^{-1}. Relevant distances and angles are also indicated.

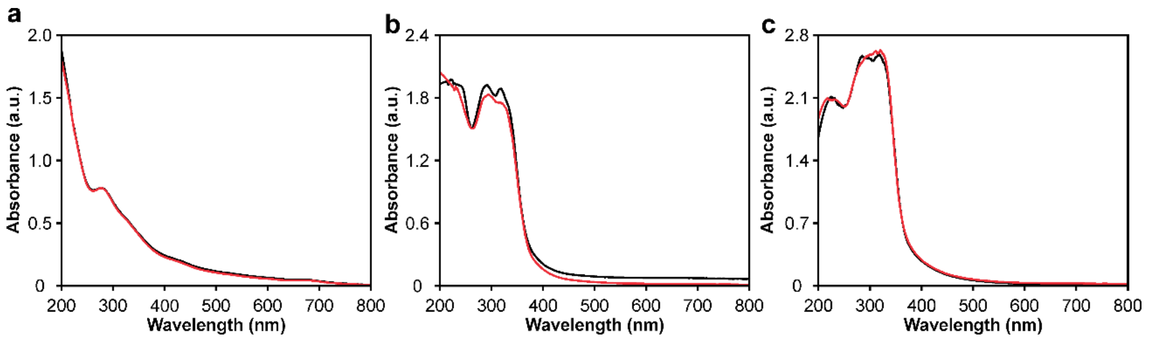

Supplementary Figure 9. Absorption spectra of isolated cuticles before (black) and after (red) TAS measurements. a, *Beta vulgaris* leaf cuticle, **b,** *Agave americana* leaf cuticle and **c,** *Capsicum annuum* fruit cuticle.

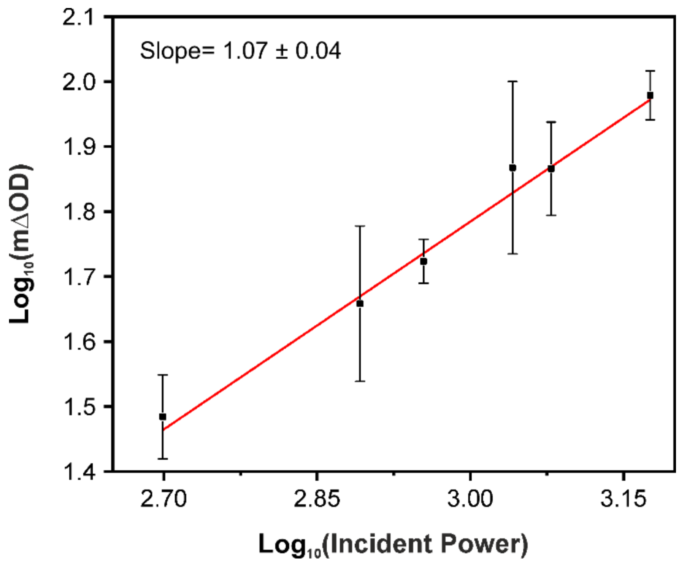

Supplementary Figure 10. Power dependency studies of *p*-coumaric acid in methanol. A linear relationship was observed indicating that the features of the absorption band recorded at ~ 360 nm can be attributed to a one-photon dynamics. Data show as mean ± 2 s.e. n=3 independent scans.

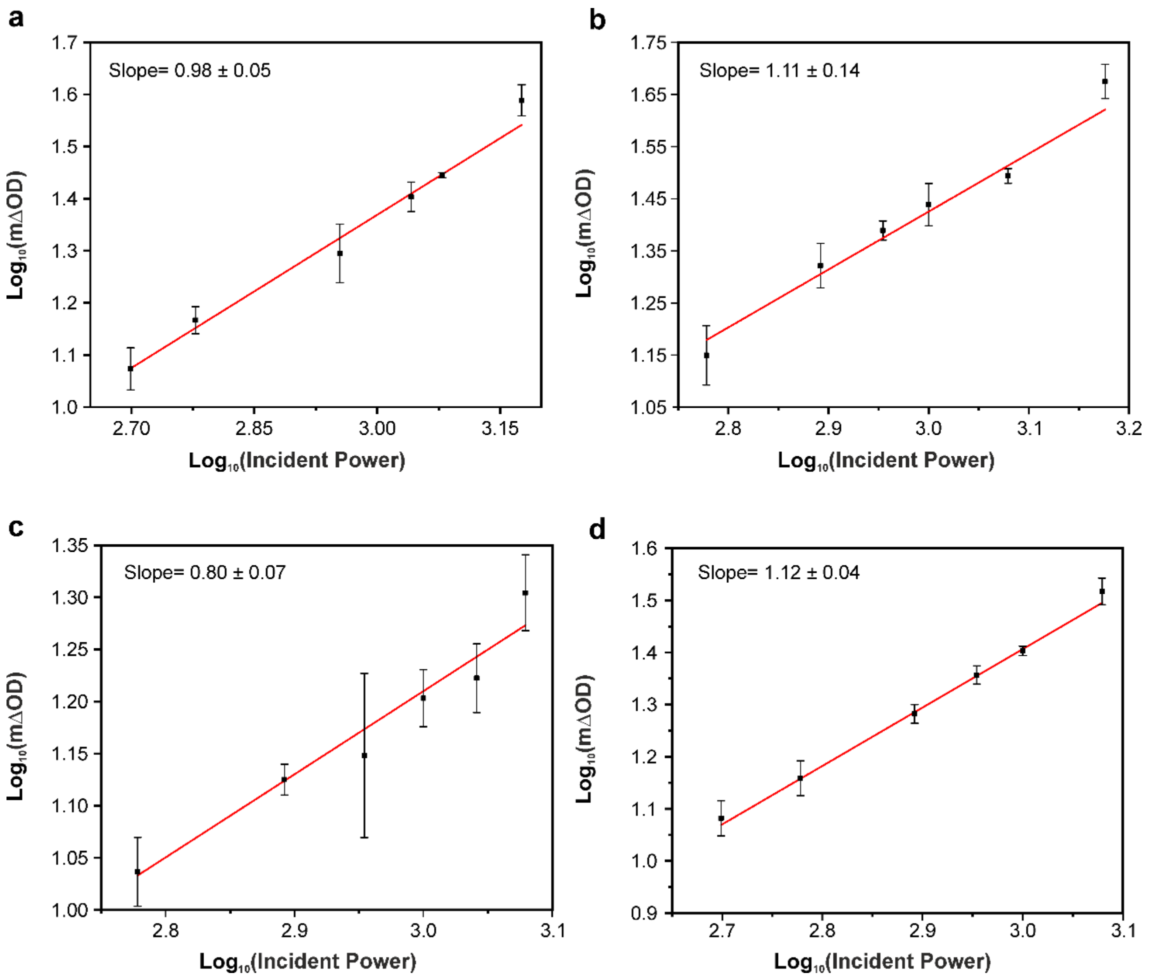

Supplementary Figure 11. Power dependency studies of powder *p*-coumaric acid and some isolated cuticles. **a,** powder *p*-coumaric acid, **b,** *Agave americana* leaf cuticle, **c,** *Hedera helix* leaf cuticle, **d,** *Brassica oleracea* leaf cuticle. A linear relationship was observed indicating that the features of the absorption band 340-450 nm can be attributed to a one-photon dynamics. Data show as mean ± 2 s.e. n=3 independent scans.

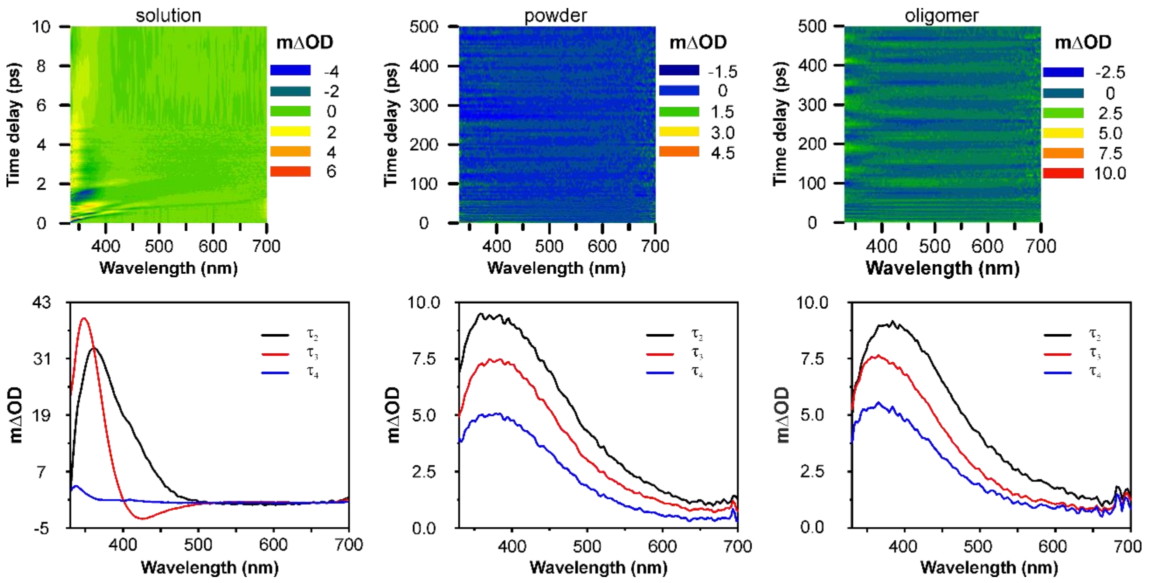

Supplementary Figure 12. Fitting residuals and evolution associated decay spectra of *p*-coumaric acid in different environments. Top, residuals, differences between measured data and those estimated from the model, at different time delays and wavelengths. Bottom, evolution associated decay spectra calculated after sequential fitting. mΔOD: changes in optical density.

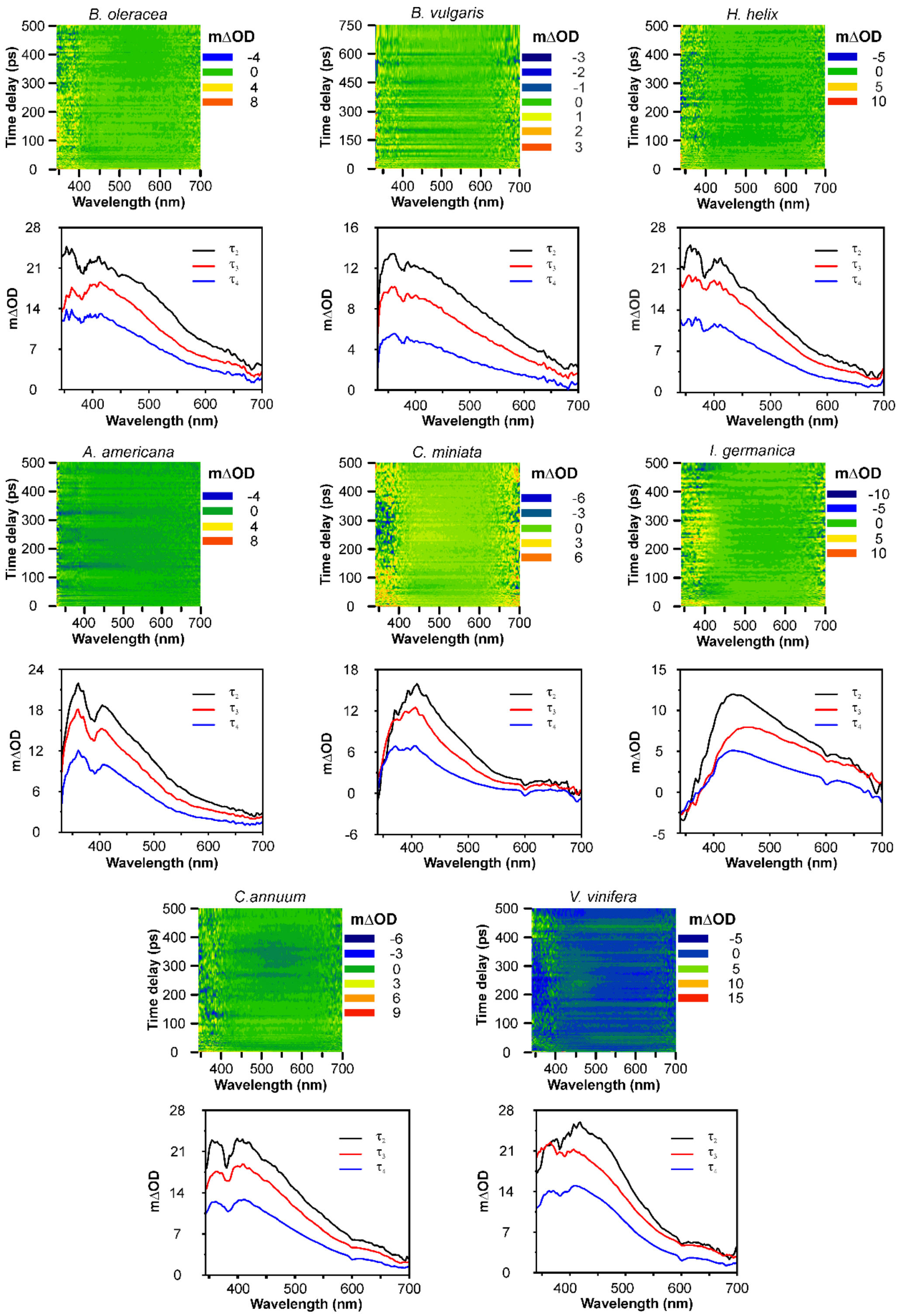

Supplementary Figure 13. Fitting residuals and evolution associated decay spectra of isolated plant cuticles. Top, residuals, differences between measured data and those estimated from the model, at different time delays and wavelengths. Bottom, evolution associated decay spectra calculated after sequential fitting. mΔOD: changes in optical density.

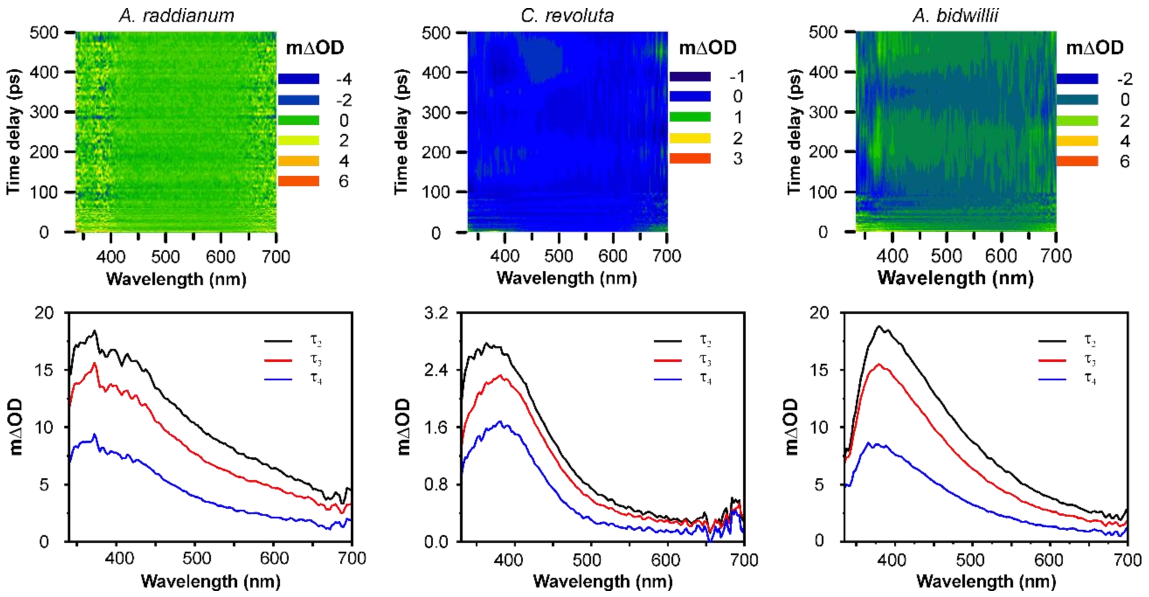

Supplementary Figure 14. Fitting residuals and evolution associated decay spectra of plant epidermises. Top, residuals, differences between measured data and those estimated from the model, at different time delays and wavelengths. Bottom, evolution associated decay spectra calculated after sequential fitting. mΔOD: changes in optical density.

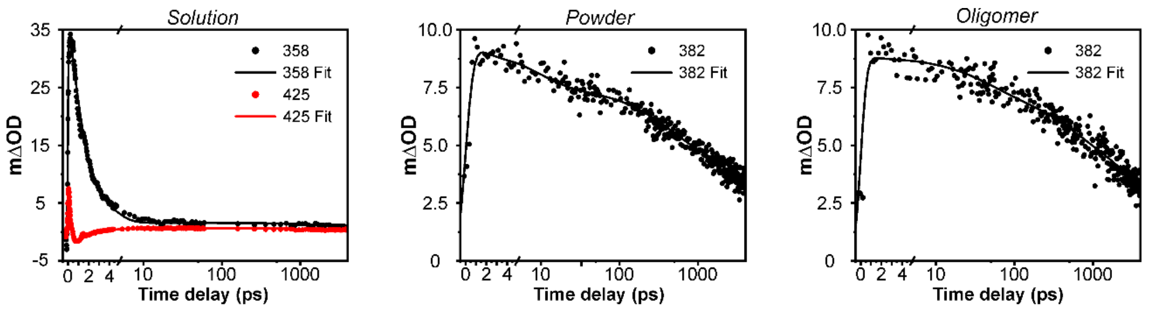

Supplementary Figure 15. Timetraces of *p*-coumaric acid in different environments. Single wavelength transient slices of the solution (358 and 425 nm), powder (382 nm) and oligomer (382 nm) of *p*-coumaric acid photoexcited with 300 nm pump pulses. Scattered points show raw data and solid lines the fitted data following a sequential model. To have a clear view of the traces, the X-axis is linear until 5 ps and log10 afterward.

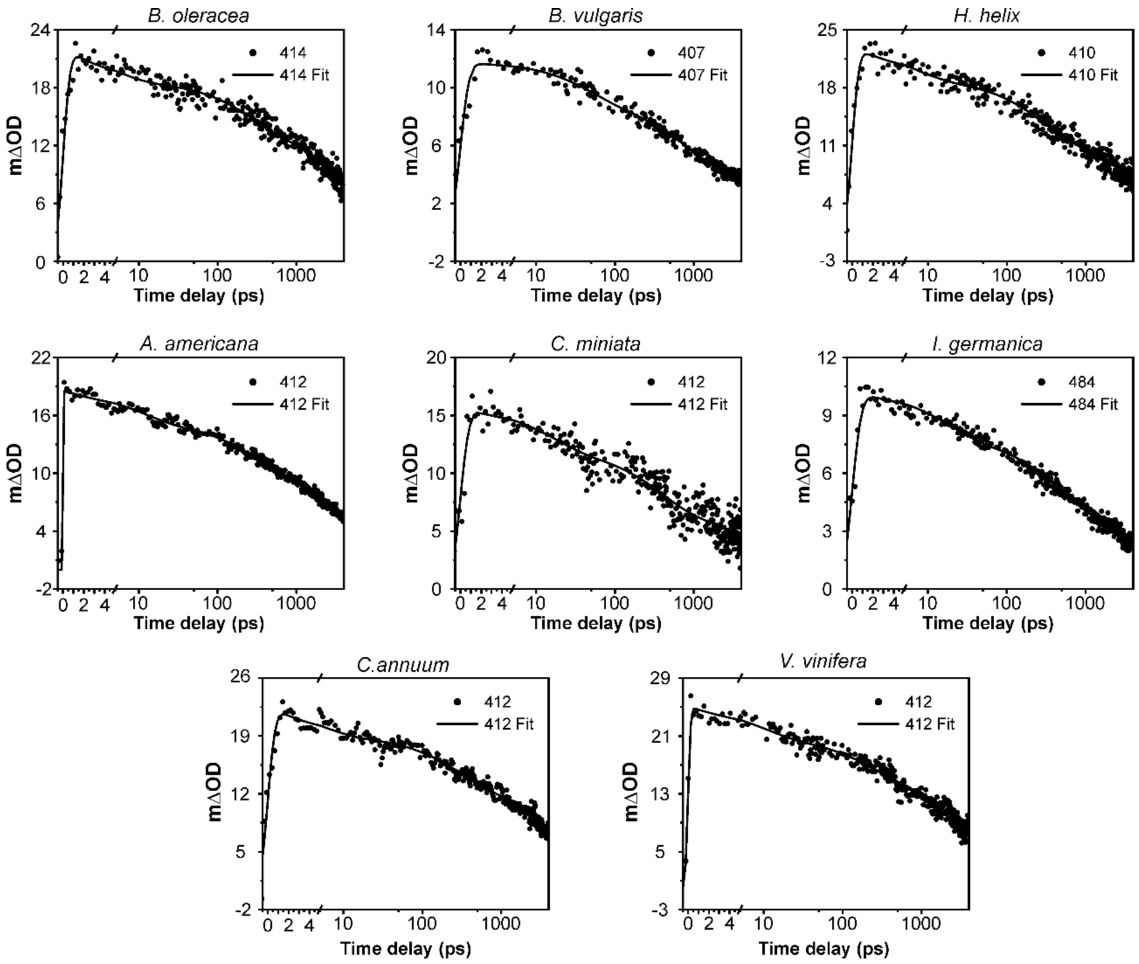

Supplementary Figure 16. Timetraces of isolated plant cuticles. Single wavelength transient slices of different species of isolated plant cuticles photoexcited with 300 nm pump pulses. Scattered points show raw data and solid lines the fitted data following a sequential model. To have a clear view of the traces, the X-axis is linear until 5 ps and log10 afterward.

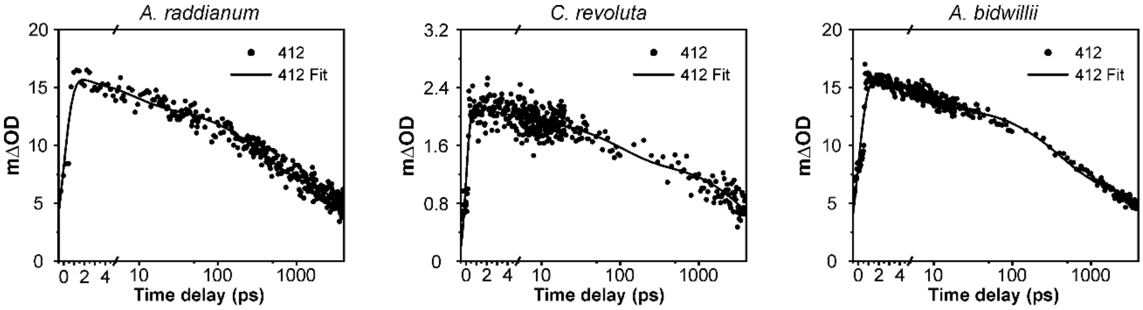

Supplementary Figure 17. Timetraces of plant epidermises. Single wavelength transient slices of different species of isolated plant cuticles photoexcited with 300 nm pump pulses. Scattered points show raw data and solid lines the fitted data following a sequential model. To have a clear view of the traces, the X-axis is linear until 5 ps and log10 afterward.

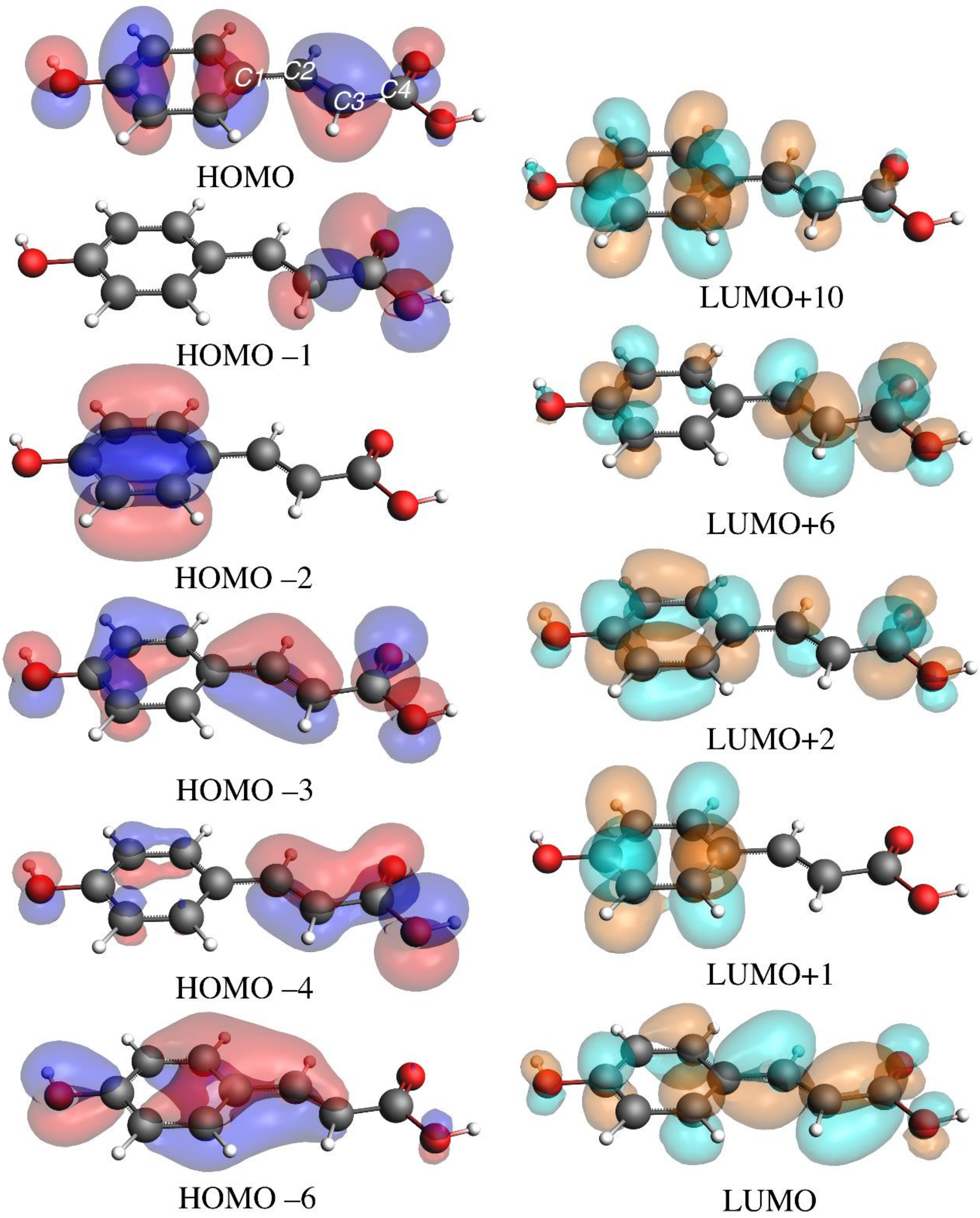

Supplementary Figure 18. Frontier orbitals selected as active space in the CASSCF calculations of the conical intersections. Red/blue and green/yellow colors are used to represent occupied and virtual orbitals, respectively.

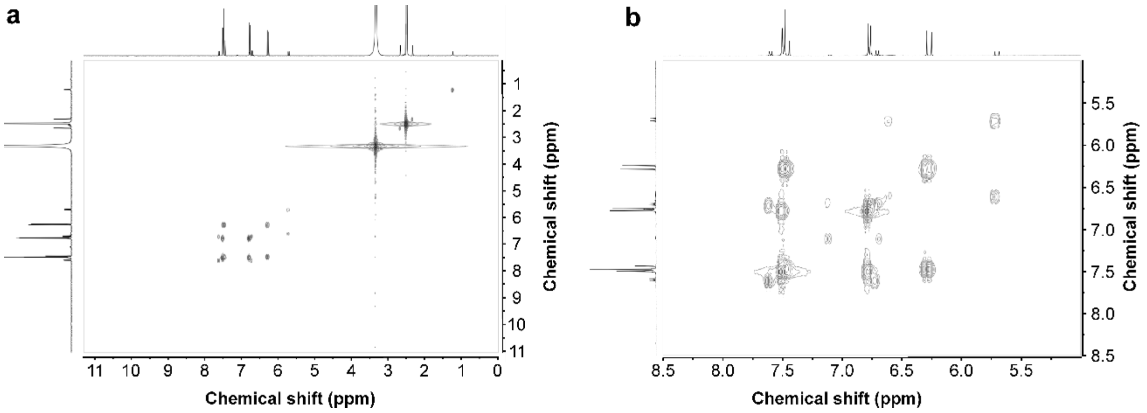

Supplementary Figure 19. 2D NMR (COSY) spectra of the *p*-coumaric solution after UV irradiation. a, full range spectrum, **b,** zoom-in of the 5.00 - 8.50 ppm region of the spectrum. Sample was irradiated at 300 nm for 5 hours.

Supplementary Figure 20. Cartesian coordinates of all the stationary points and conical intersections discussed in the main text and in the Supplementary information.

s-trans-S0

Center Number	Atomic Number	Atomic Type	Coordinates (Angstroms) X	Y	Z
1	6	0	2.642112	1.157429	-0.000065
2	6	0	1.263043	1.328333	-0.000012
3	6	0	0.388588	0.232544	0.000087
4	6	0	0.947912	-1.057770	0.000175
5	6	0	2.319885	-1.243167	0.000115
6	6	0	3.173134	-0.133426	-0.000041
7	6	0	-1.051010	0.482784	0.000059
8	6	0	-2.039841	-0.427700	-0.000033
9	6	0	-3.442713	0.007074	-0.000024
10	8	0	-4.291072	-1.038222	-0.000201
11	8	0	4.507452	-0.377626	-0.000080
12	8	0	-3.843592	1.159535	0.000135
13	1	0	0.306895	-1.933302	0.000308
14	1	0	2.747580	-2.239934	0.000167
15	1	0	3.300428	2.020985	-0.000143
16	1	0	0.857476	2.336035	-0.000039
17	1	0	-1.347529	1.530934	0.000111
18	1	0	-1.862529	-1.497489	-0.000142
19	1	0	5.004018	0.448471	-0.000481
20	1	0	-5.195302	-0.691804	-0.000180

s-trans-S1

Center Number	Atomic Number	Atomic Type	Coordinates (Angstroms) X	Y	Z
1	6	0	-2.668127	1.161195	-0.000014
2	6	0	-1.323568	1.391847	-0.000379
3	6	0	-0.363982	0.307448	-0.000526
4	6	0	-0.900598	-1.041194	-0.000534
5	6	0	-2.253329	-1.264294	-0.000146
6	6	0	-3.152454	-0.176820	0.000213
7	6	0	1.005212	0.587627	-0.000517
8	6	0	2.029260	-0.399345	-0.000365
9	6	0	3.406275	-0.024125	0.000201
10	8	0	4.266387	-1.085121	0.000307
11	8	0	-4.457287	-0.462646	0.000748
12	8	0	3.847168	1.138382	0.000605
13	1	0	-0.227325	-1.889549	-0.000847
14	1	0	-2.656177	-2.271316	-0.000088
15	1	0	-3.372050	1.987832	0.000110
16	1	0	-0.950895	2.411254	-0.000519
17	1	0	1.318511	1.627580	-0.000468
18	1	0	1.801907	-1.459608	-0.000607
19	1	0	-4.999035	0.337549	0.001082
20	1	0	5.162783	-0.722688	0.000461

s-TS-S0

Center Number	Atomic Number	Atomic Type	Coordinates (Angstroms) X	Y	Z
1	6	0	2.638100	1.013183	0.232090
2	6	0	1.370646	1.495575	0.056195
3	6	0	0.289147	0.620193	-0.283356
4	6	0	0.561269	-0.777297	-0.439328
5	6	0	1.823689	-1.266895	-0.263959
6	6	0	2.868273	-0.374818	0.077949
7	6	0	-0.990741	1.123207	-0.436106
8	6	0	-2.174888	0.354404	-0.796399
9	6	0	-3.001725	-0.132629	0.194935
10	8	0	-4.153793	-0.759257	-0.258470
11	8	0	4.066275	-0.910530	0.245354
12	8	0	-2.825764	-0.084275	1.443184
13	1	0	-0.268380	-1.427735	-0.695405
14	1	0	2.053072	-2.320284	-0.372110
15	1	0	3.460066	1.672730	0.489384
16	1	0	1.172547	2.556386	0.174664
17	1	0	-1.077845	2.204646	-0.269870
18	1	0	-2.455987	0.324296	-1.844849
19	1	0	4.730202	-0.250798	0.486868
20	1	0	-4.610025	-1.056278	0.538651

s-TS-S1

Center Number	Atomic Number	Atomic Type	Coordinates (Angstroms) X	Y	Z
1	6	0	-3.054225	-0.235305	0.158506
2	6	0	-2.660694	1.114380	-0.012522
3	6	0	-1.344302	1.412922	-0.240892
4	6	0	-0.344078	0.381200	-0.319731
5	6	0	-0.785422	-0.979652	-0.149010
6	6	0	-2.105649	-1.273406	0.088494
7	6	0	0.997424	0.703561	-0.525534
8	6	0	2.061610	-0.267897	-0.553673
9	6	0	3.276951	-0.038138	0.151856
10	8	0	3.530225	0.945162	0.869475
11	8	0	-4.328618	-0.585562	0.372916
12	8	0	4.218295	-1.009683	-0.037675
13	1	0	-0.057901	-1.781467	-0.217847
14	1	0	-2.441723	-2.297567	0.208483
15	1	0	-3.404326	1.903920	0.040698
16	1	0	-1.038423	2.447256	-0.364515
17	1	0	1.261180	1.759093	-0.597183
18	1	0	1.984712	-1.169861	-1.157450
19	1	0	-4.908998	0.186044	0.389744
20	1	0	4.996578	-0.752758	0.475372

s-cis-S0

Center Number	Atomic Number	Atomic Type	Coordinates (Angstroms) X	Y	Z
1	6	0	-1.761467	-1.222698	-0.015000
2	6	0	-2.867175	-0.368242	0.000483
3	6	0	-2.678643	1.015914	0.012553
4	6	0	-1.388640	1.525684	0.008839
5	6	0	-0.257082	0.688614	-0.005063
6	6	0	-0.476832	-0.701825	-0.017722
7	8	0	-4.097363	-0.938296	0.002563
8	6	0	1.042697	1.360701	-0.009647
9	6	0	2.341499	0.981752	-0.007980
10	6	0	2.956785	-0.350849	0.005544
11	8	0	4.302470	-0.244061	-0.002159
12	8	0	2.417662	-1.445650	0.023372
13	1	0	0.372256	-1.371586	-0.028514
14	1	0	-1.922907	-2.295481	-0.024947
15	1	0	-3.532733	1.686652	0.024073
16	1	0	-1.255931	2.603892	0.017082
17	1	0	0.930855	2.444347	-0.015902
18	1	0	3.072745	1.783296	-0.015184
19	1	0	-4.783676	-0.261847	0.012803
20	1	0	4.670386	-1.139524	0.008335

s-cis-S1

Center Number	Atomic Number	Atomic Type	Coordinates (Angstroms) X	Y	Z
1	6	0	-1.809031	-1.237812	-0.265195
2	6	0	-2.868103	-0.396302	0.069868
3	6	0	-2.646568	0.976898	0.234723
4	6	0	-1.373538	1.494485	0.068304
5	6	0	-0.279937	0.665389	-0.265748
6	6	0	-0.533486	-0.714320	-0.430284
7	8	0	-4.096874	-0.961495	0.224379
8	6	0	1.032717	1.221694	-0.415591
9	6	0	2.180835	0.436390	-0.778970
10	6	0	3.030965	-0.168819	0.230774
11	8	0	4.059199	-0.862257	-0.298451
12	8	0	2.855874	-0.079992	1.437618
13	1	0	0.277173	-1.387498	-0.689951
14	1	0	-1.993434	-2.299207	-0.392038
15	1	0	-3.471711	1.634459	0.493661
16	1	0	-1.211873	2.560133	0.199829
17	1	0	1.152368	2.297063	-0.282197
18	1	0	2.470172	0.291031	-1.819661
19	1	0	-4.746479	-0.292825	0.466172
20	1	0	4.575063	-1.238826	0.428528

s-Cl

Center Number	Atomic Number	Atomic Type	Coordinates (Angstroms) X	Y	Z
1	6	0	-1.799934	-1.234573	-0.277255
2	6	0	-2.862056	-0.399660	0.064588
3	6	0	-2.645254	0.972746	0.241884
4	6	0	-1.373863	1.496076	0.080994
5	6	0	-0.277254	0.673684	-0.259703
6	6	0	-0.526048	-0.705341	-0.436760
7	8	0	-4.089020	-0.970339	0.213176
8	6	0	1.033620	1.235715	-0.403644
9	6	0	2.184617	0.457558	-0.773236
10	6	0	3.021902	-0.174426	0.230821
11	8	0	4.055111	-0.856355	-0.303643
12	8	0	2.832870	-0.115559	1.437396
13	1	0	0.287046	-1.373439	-0.701877
14	1	0	-1.980678	-2.295403	-0.413722
15	1	0	-3.472778	1.625186	0.506122
16	1	0	-1.215873	2.561041	0.222166
17	1	0	1.149561	2.310243	-0.260545
18	1	0	2.485853	0.337789	-1.813811
19	1	0	-4.741034	-0.306047	0.460491
20	1	0	4.561830	-1.252034	0.419608

s-Cl’

Center Number	Atomic Number	Atomic Type	Coordinates (Angstroms) X	Y	Z
1	6	0	-2.559244	1.005009	0.312129
2	6	0	-1.290043	1.474528	0.135541
3	6	0	-0.242240	0.617321	-0.299742
4	6	0	-0.546464	-0.753053	-0.534978
5	6	0	-1.814361	-1.231491	-0.361395
6	6	0	-2.821598	-0.354803	0.063283
7	6	0	1.108396	1.122448	-0.451825
8	6	0	2.195384	0.402453	-0.838690
9	6	0	2.915568	-0.171698	0.215675
10	8	0	4.129622	-0.676749	-0.118035
11	8	0	-4.030746	-0.865278	0.220868
12	8	0	2.534881	-0.268749	1.379878
13	1	0	0.258311	-1.389133	-0.847710
14	1	0	-2.064262	-2.261396	-0.529846
15	1	0	-3.351693	1.653175	0.642524
16	1	0	-1.070957	2.509023	0.329444
17	1	0	1.201106	2.180546	-0.207241
18	1	0	2.455177	0.405693	-1.882424
19	1	0	-4.662720	-0.222885	0.519071
20	1	0	4.492603	-1.053100	0.674506

sp-trans-S0

Center Number	Atomic Number	Atomic Type	Coordinates (Angstroms) X	Y	Z
1	6	0	-0.960515	-1.071649	0.000582
2	6	0	-2.334946	-1.231914	0.000442
3	6	0	-3.166424	-0.107658	-0.000073
4	6	0	-2.609131	1.170511	-0.000402
5	6	0	-1.226563	1.315466	-0.000248
6	6	0	-0.373941	0.205389	0.000199
7	6	0	1.073478	0.426281	0.000272
8	6	0	2.035824	-0.509572	-0.000313
9	8	0	-4.506106	-0.327350	-0.000187
10	6	0	3.471452	-0.193651	-0.000172
11	8	0	4.351482	-1.029797	-0.000765
12	8	0	3.750624	1.130211	0.000679
13	1	0	-0.334989	-1.958281	0.001060
14	1	0	-2.785692	-2.218116	0.000761
15	1	0	-3.249139	2.049108	-0.000772
16	1	0	-0.800768	2.315090	-0.000506
17	1	0	1.387411	1.468316	0.000798
18	1	0	1.827697	-1.573759	-0.000987
19	1	0	-4.981999	0.508341	-0.000559
20	1	0	4.714069	1.205574	0.000661

sp-trans-S1

Center Number	Atomic Number	Atomic Type	Coordinates (Angstroms) X	Y	Z
1	6	0	-2.662511	1.156581	-0.000050
2	6	0	-1.316845	1.393651	0.000002
3	6	0	-0.360673	0.317966	0.000070
4	6	0	-0.886482	-1.035149	0.000151
5	6	0	-2.246031	-1.262561	0.000089
6	6	0	-3.141241	-0.184795	-0.000025
7	6	0	1.017409	0.594996	0.000036
8	6	0	2.012388	-0.403842	-0.000028
9	6	0	3.402574	-0.024476	-0.000021
10	8	0	4.254440	-1.086129	-0.000147
11	8	0	-4.457860	-0.465617	-0.000102
12	8	0	3.823140	1.132029	0.000089
13	1	0	-0.204153	-1.876471	0.000290
14	1	0	-2.648258	-2.269836	0.000133
15	1	0	-3.368900	1.982734	-0.000113
16	1	0	-0.944476	2.413653	-0.000025
17	1	0	1.354496	1.627897	0.000039
18	1	0	1.777699	-1.462299	-0.000111
19	1	0	-4.982119	0.342618	-0.000176
20	1	0	5.146424	-0.714791	-0.000124

sp-TS-S0

Center Number	Atomic Number	Atomic Type	Coordinates (Angstroms) X	Y	Z
1	6	0	-2.385614	1.013885	0.334599
2	6	0	-1.088824	1.443796	0.101210
3	6	0	-0.107079	0.556673	-0.362646
4	6	0	-0.467675	-0.779521	-0.613171
5	6	0	-1.756666	-1.219899	-0.384053
6	6	0	-2.718729	-0.323375	0.095922
7	6	0	1.243106	1.033217	-0.595431
8	6	0	2.358567	0.226337	-1.062610
9	6	0	2.769545	-0.204125	0.173956
10	8	0	3.772909	-1.044876	0.452822
11	8	0	-3.967073	-0.808065	0.295014
12	8	0	2.077135	0.314032	1.132088
13	1	0	0.287294	-1.455589	-1.001507
14	1	0	-2.047308	-2.246340	-0.577582
15	1	0	-3.137571	1.709233	0.698351
16	1	0	-0.826205	2.480268	0.294544
17	1	0	1.313742	2.126291	-0.653749
18	1	0	2.934228	0.376454	-1.963004
19	1	0	-4.548510	-0.116001	0.624198
20	1	0	3.880771	-1.044968	1.412697

sp-TS-S1

Center Number	Atomic Number	Atomic Type	Coordinates (Angstroms) X	Y	Z
1	6	0	-2.653888	1.118785	-0.004806
2	6	0	-1.333050	1.412632	-0.224286
3	6	0	-0.346012	0.377983	-0.307408
4	6	0	-0.795143	-0.980410	-0.149265
5	6	0	-2.123694	-1.267728	0.081126
6	6	0	-3.059610	-0.228975	0.151390
7	6	0	1.012372	0.681586	-0.487666
8	6	0	2.036613	-0.311656	-0.497124
9	6	0	3.293649	-0.037310	0.142224
10	8	0	4.205943	-1.039834	0.007800
11	8	0	-4.349802	-0.566848	0.349927
12	8	0	3.571366	1.002152	0.741561
13	1	0	-0.065823	-1.781820	-0.214006
14	1	0	-2.469642	-2.289431	0.193427
15	1	0	-3.391316	1.915406	0.054862
16	1	0	-1.016413	2.445715	-0.333095
17	1	0	1.315704	1.729054	-0.510211
18	1	0	1.928459	-1.248339	-1.040658
19	1	0	-4.910650	0.215670	0.365001
20	1	0	5.002203	-0.739461	0.465280

sp-cis-S0

Center Number	Atomic Number	Atomic Type	Coordinates (Angstroms) X	Y	Z
1	6	0	-2.862207	-0.369507	-0.000017
2	6	0	-2.676063	1.014126	-0.000187
3	6	0	-1.387276	1.526972	-0.000120
4	6	0	-0.255805	0.692839	0.000091
5	6	0	-0.472214	-0.698168	0.000268
6	6	0	-1.755434	-1.220322	0.000217
7	6	0	1.043985	1.365361	0.000161
8	6	0	2.340615	0.985070	0.000129
9	6	0	2.947849	-0.353154	-0.000072
10	8	0	2.408297	-1.442945	-0.000259
11	8	0	-4.092298	-0.942920	-0.000064
12	8	0	4.297673	-0.251811	-0.000055
13	1	0	0.378904	-1.365947	0.000425
14	1	0	-1.918013	-2.292515	0.000355
15	1	0	-3.531067	1.685454	-0.000365
16	1	0	-1.256306	2.605903	-0.000238
17	1	0	0.931710	2.449653	0.000255
18	1	0	3.078001	1.781030	0.000223
19	1	0	-4.775723	-0.266309	-0.000238
20	1	0	4.642412	-1.155173	-0.000219

sp-Cl

Center Number	Atomic Number	Atomic Type	Coordinates (Angstroms) X	Y	Z
1	6	0	-2.586618	1.042066	0.218218
2	6	0	-1.306967	1.462700	-0.001450
3	6	0	-0.279065	0.542756	-0.346893
4	6	0	-0.614560	-0.836894	-0.445282
5	6	0	-1.893127	-1.266659	-0.227789
6	6	0	-2.879977	-0.329380	0.105261
7	6	0	1.033549	0.982937	-0.538324
8	6	0	2.170179	0.155018	-0.868544
9	6	0	3.011675	-0.133199	0.213668
10	8	0	4.205419	-0.689674	-0.111498
11	8	0	-4.100460	-0.794038	0.310207
12	8	0	2.750717	0.054527	1.399588
13	1	0	0.175416	-1.519092	-0.691362
14	1	0	-2.166514	-2.302312	-0.293520
15	1	0	-3.363995	1.737781	0.480731
16	1	0	-1.064323	2.506094	0.089140
17	1	0	1.150432	2.057719	-0.400043
18	1	0	2.552536	0.203072	-1.872655
19	1	0	-4.717579	-0.110925	0.541555
20	1	0	4.658082	-0.854928	0.706593
